# Gene–environment interaction in externalizing problems among adolescents: evidence from the Pelotas 1993 Birth Cohort Study

**DOI:** 10.1111/jcpp.12022

**Published:** 2012-12-07

**Authors:** Christian Kieling, Mara H Hutz, Júlia P Genro, Guilherme V Polanczyk, Luciana Anselmi, Suzi Camey, Pedro C Hallal, Fernando C Barros, Cesar G Victora, Ana M B Menezes, Luis Augusto Rohde

**Affiliations:** 1 Department of Psychiatry, Hospital de Clínicas de Porto Alegre, Universidade Federal do Rio Grande do SulBrazil; 2 Department of Genetics, Universidade Federal do Rio Grande do SulBrazil; 3 Department of Basic Sciences, Universidade Federal de Ciências da Saúde de Porto AlegreBrazil; 4 Department of Psychiatry, Universidade de São PauloBrazil; 5 Postgraduate Program in Epidemiology, Universidade Federal de PelotasBrazil; 6 Department of Statistics, Hospital de Clínicas de Porto Alegre, Universidade Federal do Rio Grande do SulBrazil; 7 Postgraduate Program in Physical Education, Universidade Federal de PelotasBrazil; 8 Postgraduate Program in Health and Behavior, Universidade Católica de Pelotas, and Postgraduate Program in Epidemiology, Universidade Federal de PelotasBrazil; 9 Postgraduate Program in Epidemiology, Universidade Federal de PelotasBrazil; 10 Department of Psychiatry, Hospital de Clínicas de Porto Alegre, Universidade Federal do Rio Grande do SulBrazil; Institute for Developmental Psychiatry for Children and AdolescentsBrazil

**Keywords:** Gene–environment interaction, *DAT1*, maternal smoking, *MAOA*, childhood maltreatment, externalizing

## Abstract

**Background:**

The study of gene–environment interactions (G × E) is one of the most promising strategies to uncover the origins of mental disorders. Replication of initial findings, however, is essential because there is a strong possibility of publication bias in the literature. In addition, there is a scarcity of research on the topic originated from low- and middle-income countries (LMIC). The aim of this study was to replicate G × E hypotheses for externalizing problems among adolescents in a middle-income country.

**Methods:**

As part of the Pelotas 1993 Birth Cohort Study, 5,249 children were enrolled at birth and followed up to the age of 15 years, with an 85.7% retention rate. We sought an interaction between the homozygosity of the 10-repeat allele at the dopamine transporter (*DAT1*) gene and prenatal maternal smoking in the development of hyperactivity problems during adolescence assessed by the Strengths and Difficulties Questionnaire. We also tested for an interaction between the uVNTR polymorphism at the monoamine oxidase A (*MAOA*) and the experience of childhood maltreatment in the occurrence of conduct problems among adolescent boys.

**Results:**

Although there was a clear association between prenatal maternal smoking and hyperactivity scores in adolescence (*p* < 0.001), no main genetic or interaction effects for the *DAT1* gene were detected. Similarly, childhood maltreatment showed to be associated with conduct problems among boys (*p* < 0.001), with no observable main genetic or interaction effects for the *MAOA* gene.

**Conclusions:**

In the largest mental health G × E study performed in a LMIC to date, we did not replicate previous positive findings from the literature. Despite the presence of main environmental effects, there was no evidence of effect modification by genotype status. Additional replication efforts to measure G × E are needed to better understand the origins of mental health and illness, especially in LMIC.

## Introduction

Gene-environment interaction (G × E) studies have been one of the prevailing strategies to uncover the etiology of mental disorders over the last decade. The general notion that environmental effects are conditioned by the individual’s genetic background is not exactly new (Tabery, 2007). Empirical testing of this concept, however, required not only advances in molecular genetics, but prospective data from well-designed cohorts. After an early phase of studies reporting G × E (Rutter, Moffitt, & Caspi, 2006), more recent research has challenged the initial findings of such reports, originating a heated debate on what should be the conditions for replicability (Caspi, Hariri, Holmes, Uher, & Moffitt, 2010).

Up to now, research on G × E has almost exclusively originated from high-income countries (HIC). This is also the case for mental health studies in general (Patel, 2007), as well as those involving children and adolescents (Kieling & Rohde, 2012). One of the reasons for the lack of G × E studies from low- and middle-income countries (LMIC) could be the reduced number of prospective cohorts in these countries. Nonetheless, as 90% of all individuals under the age of 18 years live in LMIC and as many known risk factors for mental disorders tend to occur more frequently in low-resource settings, research conducted in LMIC could provide unique insights into the causes of mental health problems.

Global interest in the health of children has predominantly focused on the reduction in mortality. Although significant advances have been achieved in terms of promoting child survival, a renewed commitment to global child health is needed (Victora, 2012). The burden that mental health problems impose on children and adolescents worldwide justifies their inclusion as one of the priorities in health research (Kieling et al., 2011).

The three birth cohorts in the city of Pelotas, Brazil, are examples of longitudinal studies in a middle-income setting that have contributed to the understanding of risk and protective factors associated with child health (Santos et al., 2011; Victora & Barros, 2006; Victora et al., 2008). All live births in the years of 1982, 1993 and 2004 that occurred in Pelotas (around 320,000 inhabitants, located in the southern part of Brazil) have been included in these studies. Mental health information has also been collected, and more recently DNA samples were obtained for the 1993 cohort. This has allowed the completion of the largest mental health G × E study conducted in a LMIC, for which we here present the data on externalizing problems among adolescents.

Externalizing problems are characterized by a wide range of behaviors, including features usually observed in the diagnoses of attention deficit/hyperactivity disorder (ADHD), oppositional defiant disorder, and conduct disorder (CD). Although some degree of overlap in terms of psychopathological presentation exists, there are also features specific to each of these disorders (Bezdjian et al., 2011), which can be a result of different pathophysiological processes.

Attention deficit/hyperactivity disorder affects approximately 5% of children and adolescents worldwide (Polanczyk, de Lima, Horta, Biederman, & Rohde, 2007). Despite the high heritability estimates (around 80%) and intense research efforts, uncovering the biological underpinnings of ADHD is still an unfulfilled promise in the field of molecular genetics, with candidate genes exhibiting only small effect sizes (Genro, Kieling, Rohde, & Hutz, 2010; Gizer, Ficks, & Waldman, 2009). Nongenetic factors in the etiology of ADHD have also been extensively investigated: environmental factors such as maternal use of tobacco, alcohol, and other drugs during pregnancy; nutritional factors; and psychosocial adversities have all been studied with controversial results (Thapar, Cooper, Jefferies, & Stergiakouli, 2012). A plausible explanation for inconsistencies in studies of both genetic and environmental factors is the occurrence of G × E (Thapar, Harold, Rice, Langley, & O’Donovan, 2007). To date, however, only a reduced number of ADHD G × E findings were replicated. The dopamine transporter gene (*DAT1*) is one of the most intensely studied, having already been assessed in interaction with psychosocial factors (two positive studies vs. one negative study); prenatal cigarette exposure (2 vs. 2); and prenatal alcohol exposure (1 vs. 2) (Nigg, Nikolas, & Burt, 2010).

The occurrence of CD has also been documented in different cultures (Canino, Polanczyk, Bauermeister, Rohde, & Frick, 2010). Heritability estimates for CD are usually somewhat lower than those for ADHD and suggest that individual symptoms may be differentially heritable. The presence of CD in childhood and adolescence has been linked to the development of antisocial behavior later in life (Moffitt, 1993). In fact, one of the pioneering studies of G × E included CD as part of the composite index of antisocial behavior used as the primary outcome measure (Caspi et al., 2002). By assessing the interaction between childhood maltreatment and a functional polymorphism in the gene encoding the enzyme monoamine oxidase A (*MAOA*), this study proposed that maltreated males with the low *MAOA* activity genotype were more likely than nonmaltreated males with this genotype to develop CD; in contrast, among males with high MAOA activity, maltreatment did not confer significant risk for CD. This finding has been replicated by some, but not all, subsequent studies, and a meta-analysis including seven other samples confirmed the initial results, even with the exclusion of the original report (Taylor & Kim-Cohen, 2007). Since then, new reports have been published, using a variety of designs and presenting mixed results.

The aim of the present study was to assess the presence of G × E in relation to the occurrence of externalizing behavior among adolescents in a middle-income country. Specifically, we used data from the Pelotas 1993 Birth Cohort Study to test for an interaction between *DAT1* genotype and smoking during pregnancy in the development of inattention and hyperactive problems, and for an interaction between *MAOA* genotype and childhood maltreatment in the occurrence of conduct problems. Our *a priori* hypotheses were that we would replicate G × E findings originally reported in studies conducted in HIC, with (a) adolescents homozygous for the 10-repeat allele in the *DAT1* gene showing higher levels of ADHD symptoms when maternal smoking was present during pregnancy, and (b) male adolescents with low activity *MAOA* genotype presenting higher levels of CD when childhood maltreatment was reported.

## Methods

Participants of this study were individuals followed in the Pelotas 1993 Birth Cohort Study, as described in detail elsewhere (Victora et al., 2008). In brief, all children born alive in the city during the year of 1993 (*N* = 5,265) were eligible. Only 16 mothers could not be interviewed at baseline or refused to participate in the study. Follow-up visits were scheduled at multiple time points (the last one in 2008) and included assessments of mental health problems and risk factors in interviews with the child/adolescent and the primary caregiver. The data used in the analyses here were collected at the perinatal and at the 11 and 15 years assessments.

For recruitment, daily visits were conducted in all five hospitals in the city of Pelotas from 1 January to 31 December 1993. Mothers were interviewed on a variety of topics on perinatal health status. Information on smoking during each trimester of pregnancy was collected and mothers were classified as smokers if they reported the habit in any trimester. Data were also obtained on the use of alcohol during pregnancy (yes/no), skin color of the child, maternal education (number of years attending school grouped in three strata), and monthly family income (measured in number of minimum wages, a standard unit in Brazil valued around USD 60 in 1993). Additional visits to subsamples of the entire cohort occurred in the first years of life (Victora et al., 2008)

The 2004 visit (at age 11) attempted to include all individuals originally assessed at birth, with a follow-up rate of 87.5%. Child mental health data were collected using the validated Brazilian Portuguese version of the Strengths and Difficulties Questionnaire (SDQ) (Goodman, 1997; Fleitlich-Bilyk & Goodman, 2004). The hyperactivity and conduct subscales of the SDQ (both ranging from 0 to 10), collected with the primary caregiver, were used as outcome measures for the present study. Data on maternal mental health status were obtained using the Brazilian Portuguese validated version of the Self-Report Questionnaire (SRQ –Mari & Williams, 1986).

In 2008 (at age 15), 85.7% of all children originally included in the baseline visit were assessed. Adolescents answered a confidential form that included seven questions about domestic violence, physical, and sexual abuse. Following the strategy adopted by Caspi et al. (2002), individuals were classified into three categories: no maltreatment, probable maltreatment (one positive answer), and severe maltreatment (two or more positive answers). Information on mental health (hyperactivity and conduct SDQ subscales) was collected using the same strategy as in 2004.

DNA was obtained in the last visit using saliva samples and isolated with the Oragene DNA kit (DNA Genotek Inc, Kanata, ON, Canada) according to the manufacturer’s instructions. The 40-bp variable number of tandem repeats (VNTR) at the 3 untranslated region (UTR) of *DAT1* was amplified using the polymerase chain reaction (PCR) with protocols previously described (Roman et al., 2001). Genotype groups were defined by the presence or absence of two copies of the 10-repeat allele. The MAOA-uVNTR was amplified by PCR using primers and methods previously described by Guimarães et al. (2009). High and low activity groups were defined using the same criteria as in the original study by Caspi et al. (2002). Analyses including the *MAOA* gene were restricted to boys because of the uncertainty of activity status among girls (due to X-chromosome inactivation) and to allow for comparisons with most of the published results.

To examine whether genotypes moderated the effect of environment on SDQ measures, linear regression analyses were used (with preference to the data collected at age 15). Logistic regression analyses were performed to confirm results using dichotomized outcome measures. Caseness was operationally defined as having an SDQ score above the 95th percentile for hyperactivity or conduct problems.

Conceptually defined potential confounders included in the analyses were gender, skin color, maternal prenatal use of tobacco and alcohol, maternal education and mental health, and family income. Statistical analyses were performed using PASW Statistics, version 20 (SPSS Inc., Chicago, IL). This study was approved by the Institutional Review Board of the School of Medicine, Universidade Federal de Pelotas (UFPel). Informed consent was obtained from the primary caregiver.

## Results

A total of 4,101 adolescents provided saliva DNA samples for current analyses; 48.9% were boys. Around two thirds (66.2%) were classified as having white skin color. One third (33.2%) of the mothers stated having smoked and 5.2% reported using alcohol during pregnancy. Mean family monthly income was 4.2 minimum wages [standard deviation (*SD*): 5.7]. Maternal education as measured by the number of years of school attendance was distributed as follows: 0–4 years, 24.5%; 5–8 years, 48.2%; and 9 years or more 27.4%. Screening for maternal psychopathology using SRQ was positive in 30.8% of the cases. No evidence of maltreatment was found in 67.0% of the individuals; 18.3% and 14.7% of the adolescents were classified as suffering probable and severe maltreatment, respectively. Both SDQ scores declined from ages 11 to 15: for hyperactivity mean scores decreased from 4.3 (*SD*: 3.1) to 3.8 (3.1); and for conduct scores from 2.5 (2.3) to 2.3 (2.3). Table 1 presents the sample characteristics according to genotype group.

**Table 1:** Sample characteristics according to genotype groups

	*DAT1* genotype		*MAOA* genotype	
		
	other (*n *= 2,066)	10/10 (*n *= 2,027)	low activity (*n *= 742)	high activity (*n *= 1,256)
Men	48.7%	49.0%	100%	100%
White skin color	64.8%	67.5%	62.0%	71.1%
Maternal education (years)				
0–4	25.0%	23.9%	23.2%	25.6%
5–8	48.3%	48.1%	47.7%	49.8%
9 or more	26.8%	28.0%	29.1%	24.7%
Maternal alcohol	4.6%	5.8%	4.7%	4.8%
Maternal tobacco	33.2%	33.2%	29.8%	34.6%
Family income (minimum wages)	4.3 (5.8)	4.2 (5.5)	4.2 (5.5)	4.3 (5.9)
Maternal psychopathology	30.4%	31.2%	30.5%	32.2%
Childhood maltreatment				
No	67.3%	66.6%	74.2%	73.2%
Probable	18.1%	18.6%	16.6%	16.6%
Severe	14.6%	14.8%	9.2%	10.2%
SDQ hyperactivity (age 11)	4.3 (3.1)	4.3 (3.1)	4.9 (3.0)	4.8 (3.1)
SDQ hyperactivity (age 15)	3.9 (3.1)	3.8 (3.1)	4.3 (3.2)	4.2 (3.1)
SDQ conduct (age 11)	2.5 (2.4)	2.5 (2.3)	2.7 (2.4)	2.6 (2.3)
SDQ conduct (age 15)	2.3 (2.3)	2.3 (2.2)	2.2 (2.2)	2.2 (2.2)

Categorical data presented as percentages; continuous variables as mean (standard deviation).

*DAT1*, dopamine transporter gene; *MAOA*, monoamine oxidase A gene; SDQ, Strengths and Difficulties Questionnaire.

**Figure 1:**
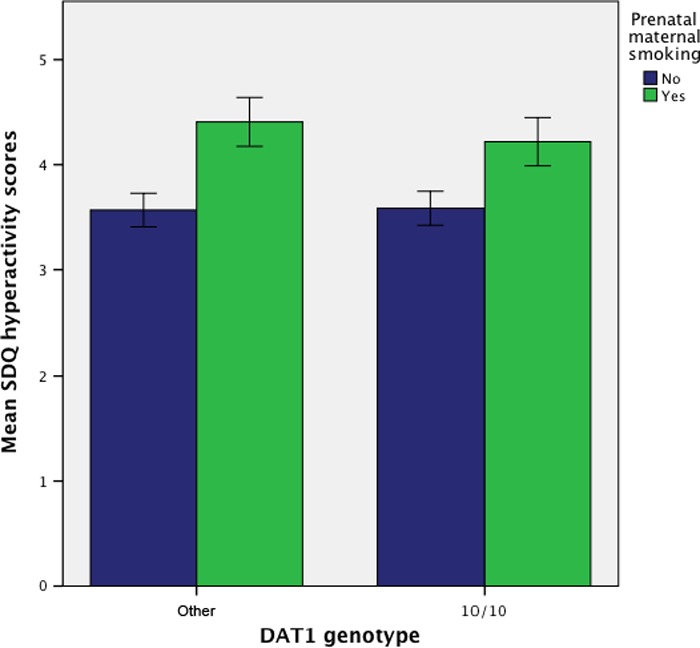
SDQ hyperactivity scores at age 15 according to genotype status and maternal prenatal smoking. Error bars represent 95% confidence intervals. SDQ, Strengths and Difficulties Questionnaire; *DAT1*, dopamine transporter gene

Genotype for the *DAT1* was successfully defined for 4,093 adolescents, 49.5% of them being homozygous for the 10-repeat allele. Regression analyses showed evidence for an association between maternal smoking during pregnancy and SDQ hyperactivity scores at age 15 (*p* < 0.001). No evidence, however, was found for an association between genotypic groups and SDQ hyperactivity scores (*p* = 0.257) or for a different pattern of dose-response relationship between maternal smoking and hyperactivity according to *DAT1* genotype group (test for interaction *p* = 0.309) (Figure 1). Inclusion of all potential confounders in the model showed that gender (*p* < 0.001), skin color (*p* = 0.027), maternal mental health (*p* < 0.001), use of alcohol (*p* = 0.012), and family income (*p* = 0.001) were associated with the outcome, but did not change the main results. Analyses using the 11 years SDQ hyperactivity measures or the dichotomized variable as outcome exhibited similar results (data available upon request).

**Figure 2:**
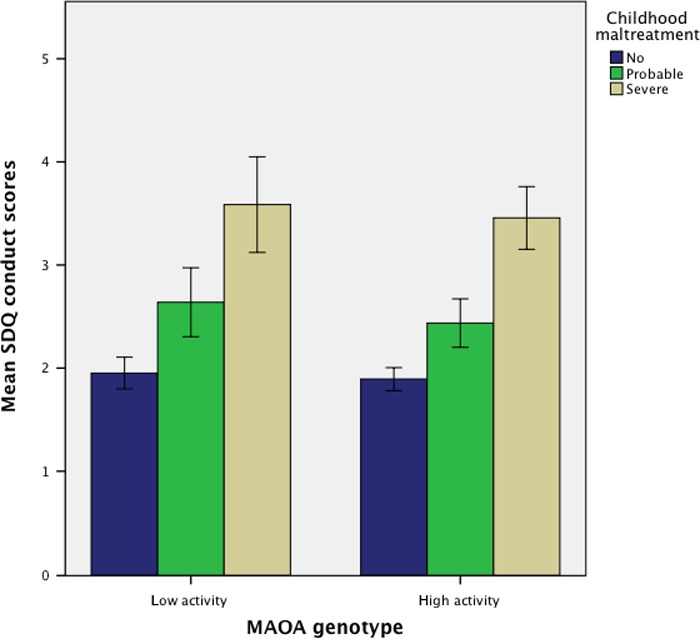
SDQ conduct scores at age 15 according to genotype status and childhood maltreatment status. Error bars represent 95% confidence intervals. SDQ, Strengths and Difficulties Questionnaire; *MAOA*, monoamine oxidase A gene

Genotype for the *MAOA* was successfully characterized for 1,998 boys, 37.1% of them possessing a low activity allele. In a similar fashion, regression analyses suggested the presence of an association between childhood maltreatment and SDQ conduct scores (*p* < 0.001). No evidence, however, was found for an association between high and low activity genotypic groups and SDQ conduct scores (*p* = 0.223) or for a different pattern of dose-response relationship between maltreatment and conduct according to *MAOA* genotype group (test for interaction *p* = 0.823) (Figure 2). Inclusion of all potential confounders in the model showed that skin color (*p* = 0.001), maternal education (*p* = 0.010) and mental health (*p* < 0.001), prenatal exposure to alcohol (*p* = 0.001) and tobacco (*p* = 0.007), and family income (*p* = 0.023) were associated with the outcome, but did not change the main results. As for the previous analysis, using the dichotomized variable as outcome did not change the pattern of results (data available upon request).

## Discussion

This study assessed candidate G × E in relation with the occurrence of externalizing behavior during adolescence. We attempted to replicate some of the positive G × E findings from the HIC literature in a large population-based birth cohort in a middle-income setting. Although there was a clear association of environmental risk factors with higher levels of externalizing problems (results remained unchanged even after including all *a priori* defined covariates in the regression models), neither main genetic nor G × E effects were detected.

The growing literature on G × E for ADHD has assessed a variety of genetic and environmental risk factors, but with few reports replicating the same selection of variables and designs (for a review, see Nigg et al., 2010). The first study to evaluate the interaction between *DAT1* polymorphisms and smoking during pregnancy in the etiology of ADHD was published by Kahn, Khoury, Nichols, and Lanphear (2003). They assessed 161 children at 5 years of age; information on prenatal smoking was retrospectively collected when the child was 6 months old. Results indicated that children both homozygous for the 10-repeat allele and with prenatal smoke exposure had higher levels of hyperactive-impulsive, but not inattentive symptoms. It is possible that the decline in motor symptoms in late childhood and in adolescence was responsible for the lack of replication of findings in the current sample. A subsequent study failed to identify an interaction between *DAT1* gene (investigating VNTRs in the 3′UTR and also in intron 8) and environment (defined as smoking at least 20 cigarettes per day) in two samples (*n* = 396 children aged 5–15 and their parents) from child behavior clinics in the United Kingdom and Taiwan (Brookes et al., 2006). A twin-study by Neuman et al. (2007) (140 cases and 692 controls) pointed to the existence of G × E between *DAT1* and prenatal smoking, but in this report the risk genotype was the presence of a 9-repeat allele (with an odds ratio of 2.9 for having a DSM-IV combined subtype diagnosis). Becker, El-Faddagh, Schmidt, Esser, and Laucht (2008) found an interaction between *DAT1* and prenatal smoking exposure, a finding that was significant only for males; in the present study, no such specific effects were detected (both including gender as a covariate and stratifying analyses for boys and girls). Langley et al. (2008) assessed G × E for multiple genetic markers and risk factors in a clinical sample of 266 children, showing no evidence of interaction between *DAT1* and prenatal smoking.

Methodological differences in terms of design, sample composition, definition of risk factors, selection of genetic markers, and outcome measure may explain why results have been heterogeneous. Interestingly, novel genetically sensitive research designs have questioned the role of maternal smoking during pregnancy in the etiology of ADHD, as the association between the two variables might be a consequence of an inherited effect (Thapar et al., 2009). Specific gene-environment correlations (rGE) – in which exposure to environmental experiences depends on an individual’s genotype – remain to be tested.

Research on the interaction between maltreatment and *MAOA* genotype has been more consistent. The landmark study by Caspi et al. (2002) provided epidemiological evidence that the effect of maltreatment on the development of antisocial behavior (including CD during adolescence) was moderated by *MAOA* activity group genotype. Replication studies had mixed results. The one existing meta-analysis (Taylor & Kim-Cohen, 2007) confirmed the existence of G × E as correlation coefficients between antisocial behavior and maltreatment varied according to *MAOA* genotype status (0.30; 95%IC 0.24–0.36 for the low activity group and 0.13; 95%IC 0.09–0.17 for the high activity group).

Again, heterogeneity in the definition of antisocial behavior across different studies might be one of the explanations for mixed results. The wide age range assessed in different samples might also be one of the reasons for lack of consistency, as presentation and persistence of antisocial behavior tend to vary across the lifespan (Moffitt, 1993). Another source of discrepancy among studies might be a ceiling effect of high levels of maltreatment, leaving no room for variability according to genotype status, as suggested by Weder et al. (2009).

Strengths of the present study include the follow-up of a large number of individuals in a population-based cohort from birth until mid-adolescence, with a low attrition rate. Multiple strategies have been developed by the local research team to find study participants, including media advertisements and internet searches. A major limitation of the present report is the use of a screening instrument as the outcome variable. The SDQ, however, was sufficiently robust for the detection of main environmental effects, and it has been already used in other G × E studies assessing externalizing behavior with positive results (Enoch, Steer, Newman, Gibson, & Goldman, 2010). A validation study compared SDQ with DSM-IV diagnoses in a subsample of the Pelotas 1993 Birth Cohort Study, showing that the former had moderate predictive power, with an area under the curve in ROC analyses of 0.74 (95% CI 0.68–0.81) (Anselmi, Fleitlich-Bilyk, Menezes, Araújo, & Rohde, 2010).

Self-report has been originally suggested to be a viable proxy measure for cotinine levels among women who smoke during pregnancy, but recent data have challenged this assumption in Scotland (Shipton et al., 2009). In this sense, assessment of prenatal smoking ideally would have been conducted with objective methods prospectively during pregnancy. Although a potential risk of underreport exists, a recent joint analysis of the Pelotas and Avon Longitudinal Study of Parents and Children cohorts indicated that maternal prenatal smoking in the Brazilian city was twice as frequent as in the British cohort (Brion et al., 2010). In addition, smoking during gestation in the 1993 cohort was strongly associated with low birth weight and other outcomes in infancy for which smoking is a well-known risk factor (Horta, Victora, Menezes, Halpern, & Barros, 1997). The fact that information on maltreatment was retrospectively collected at the same time as data on adolescent externalizing behavior makes the G × E analysis for conduct problems a cross-sectional one and raises the possibility of information bias.

Replication of findings has been continuously recognized as *condicio sine qua non* for the acceptance of G × E hypotheses (Moffitt, Caspi, & Rutter, 2005). Although the exact strategy to compare studies (broad vs. narrow replications) is still in debate (Caspi et al., 2010), the risk of false positives casts no doubt on the need to further substantiate G × E effects in psychopathology (Asherson & Price, 2012). Publishing negative findings is essential, because positive G × E results are reported in 96% of novel studies but only among 27% of the replication attempts, and because those replications with positive results tend to have smaller average sample sizes in comparison to the negative ones – thus providing evidence of strong publication bias in the G × E literature (Duncan & Keller, 2011).

The fact that isolated nature or nurture effects cannot account for the occurrence of mental disorders makes gene–environment interplay a compelling model to better understand the origins of mental health and illness. The search for specific G × E, however, still represents a complex and unfinished task, as heterogeneity in terms of definition of variables of interest, research designs, and samples assessed have to be taken into account when interpreting results or planning future studies. In this sense, replicating G × E studies in LMIC may not only shed light on the etiology of mental health problems where nine out of ten children and adolescents live, but also provide valuable information on the validity of proposed models.

**Key points**

- Measuring G × E has received an increasing attention in the mental health literature. Initial findings, such as *MAOA* x childhood maltreatment in the development of antisocial behavior or *DAT1* × maternal prenatal smoking, have not always been replicated.
- The literature on G × E has predominantly originated in HIC, and there is a possible publication bias, with fewer negative findings than expected being published.
- In the largest mental health G × E in a LMIC to date, we replicated the expected associations between environmental risk factors and externalizing problems (maternal smoking for hyperactivity and childhood maltreatment for conduct).
- However, we did not observe any evidence of main genotypic or interaction effects of the *DAT1* and *MAOA* genotype relevant to the occurrence of externalizing problems during adolescence.

## Correspondence

Luis Augusto Rohde, MD, PhD, Department of Psychiatry, Hospital de Clínicas de Porto Alegre, Universidade Federal do Rio Grande do Sul. Rua Ramiro Barcelos, 2530 – 400N. Porto Alegre 90035-009 RS, Brazil; Email: lrohde@terra.com.br
